# Field performance of a low-cost sensor in the monitoring of particulate matter in Santiago, Chile

**DOI:** 10.1007/s10661-020-8118-4

**Published:** 2020-02-10

**Authors:** Matías Tagle, Francisca Rojas, Felipe Reyes, Yeanice Vásquez, Fredrik Hallgren, Jenny Lindén, Dimitar Kolev, Ågot K. Watne, Pedro Oyola

**Affiliations:** 1 Centro Mario Molina Chile, Antonio Bellet 292, Providencia, Santiago, Chile; 2 IVL Swedish Environmental Research Institute, Aschebergsgatan 44, Gothenburg, Sweden; 3 RISE Acreo, Research Institutes of Sweden, Lindholmspiren 7 A, Gothenburg, Sweden; 4 Environment Administration, City of Gothenburg, Karl Johansgatan 23, Gothenburg, Sweden

**Keywords:** Citizen science, Reproducibility, Relative humidity, SDS011

## Abstract

Integration of low-cost air quality sensors with the internet of things (IoT) has become a feasible approach towards the development of smart cities. Several studies have assessed the performance of low-cost air quality sensors by comparing their measurements with reference instruments. We examined the performance of a low-cost IoT particulate matter (PM_10_ and PM_2.5_) sensor in the urban environment of Santiago, Chile. The prototype was assembled from a PM_10_–PM_2.5_ sensor (SDS011), a temperature and relative humidity sensor (BME280) and an IoT board (ESP8266/Node MCU). Field tests were conducted at three regulatory monitoring stations during the 2018 austral winter and spring seasons. The sensors at each site were operated in parallel with continuous reference air quality monitors (BAM 1020 and TEOM 1400) and a filter-based sampler (Partisol 2000i). Variability between sensor units (*n* = 7) and the correlation between the sensor and reference instruments were examined. Moderate inter-unit variability was observed between sensors for PM_2.5_ (normalized root-mean-square error 9–24%) and PM_10_ (10–37%). The correlations between the 1-h average concentrations reported by the sensors and continuous monitors were higher for PM_2.5_ (*R*^2^ 0.47–0.86) than PM_10_ (0.24–0.56). The correlations (*R*^2^) between the 24-h PM_2.5_ averages from the sensors and reference instruments were 0.63–0.87 for continuous monitoring and 0.69–0.93 for filter-based samplers. Correlation analysis revealed that sensors tended to overestimate PM concentrations in high relative humidity (RH > 75%) and underestimate when RH was below 50%. Overall, the prototype evaluated exhibited adequate performance and may be potentially suitable for monitoring daily PM_2.5_ averages after correcting for RH.

**Electronic supplementary material:**

The online version of this article (10.1007/s10661-020-8118-4) contains supplementary material, which is available to authorized users.

## Introduction

Particulate matter (PM) air pollution is currently the leading environmental risk factor for premature death (Cohen et al. 2017; Gakidou et al. 2017). Global estimates indicate diseases resulting from long-term exposure to PM account for 4 to 9 million deaths annually (Burnett et al. 2018; Stanaway et al. 2018). Particles with an aerodynamic diameter (*d*_*a*_) smaller than 10 μm (μm), collectively identified as PM_10_, are potentially harmful since they can be inhaled. However, the greatest concern relates to the fine fraction, or particles with a d_a_ ≤ 2.5 μm (PM_2.5_). Although the mechanisms remain unclear, there are indications that PM_2.5_ can penetrate the alveolar epithelium, enter the bloodstream and migrate to various organs in the human body (Nakane, 2012; Li et al. 2019).

While the air concentration of PM_2.5_ varies spatially and temporally (Karagulian et al. 2015; Cheng et al. 2016), a considerable proportion of the world’s population (91%) reside in cities where the PM_2.5_ concentrations exceed suggested thresholds (World Health Organization, 2018). Given the health risk of fine particles, the ambient air concentrations of PM are widely monitored by public agencies at so-called regulatory air quality stations. These sites are equipped with instrumentation that performs standard reference methods, namely beta attenuation monitors (BAM), tapered element oscillating microbalances (TEOM), and filter-based gravimetric samplers. These scientific-grade devices are characterized as being large and expensive, among other features that hinder the expansion of air quality monitoring networks (Borrego et al. 2015).

Developments in sensor technology over the last decade have led to the emergence of miniature, commercially available, low-cost devices (less than 100 US dollars) for the surveillance of air pollution (Kumar et al. 2015). The appearance of these inexpensive sensors has resulted in a change in the monitoring paradigm, with a shift from the current governmental model towards the establishment of community-based monitoring networks (Snyder et al. 2013; Rai et al. 2017; Morawska et al. 2018). The ease with which citizens can acquire air quality monitors has led to the development of multiple crowdsourced projects that aim to increase the density of monitoring networks in regions that currently lack air quality monitoring equipment (Thompson, 2016; Castell et al. 2017; Chen et al. 2017).

Despite this progress in sensor development, systematic research on the reliability and accuracy of the measurements obtained using new devices has just begun to be conducted. Several experiments have examined the performance of commercially available, low-cost sensors, including laboratory tests (Wang et al. 2015; Manikonda et al. 2016; Papapostolou et al. 2017) and field comparisons against reference methods at regulatory air quality monitoring stations (Holstius et al. 2014; Zikova et al. 2017; Feinberg et al. 2018; Kuula et al. 2019). Although a small number of models have indicated adequate correlations between the sensors and reference instruments, there is consensus that the current generation of low-cost sensors needs further improvements to achieve the accuracy of reference monitors (Budde et al., 2014; Hall et al. 2014; Clements et al. 2017). Since most low-cost PM sensors calculate concentrations based on the principle of light scattering, environmental variables such as temperature and relative humidity (RH) may significantly bias their measurements. Research conducted to date suggests the performance of the sensors decreases in environments with RH ≥ 75%, mainly due to hygroscopic growth of the particles after condensation of water droplets (Zheng et al. 2018; Jayaratne et al. 2018; Crilley et al. 2018).

Despite these limitations, the integration of Wi-Fi microchips and internet of things (IoT) technology in recent years has improved the monitoring potential of low-cost sensors and provides an opportunity to achieve significant progress in the development of smart cities (Alvear et al. 2018). In this study, we examined the field performance of a custom-built IoT air quality sensor prototype, which was developed within the framework of the citizen science project LoV-IoT, (http://www.loviot.se). Our objective was to provide the first examination of the suitability of a low-cost sensor for the monitoring of PM_10_ and PM_2.5_ in the urban environment of Santiago, Chile.

## Methods

### Sensor assembly

**Fig. 1:**
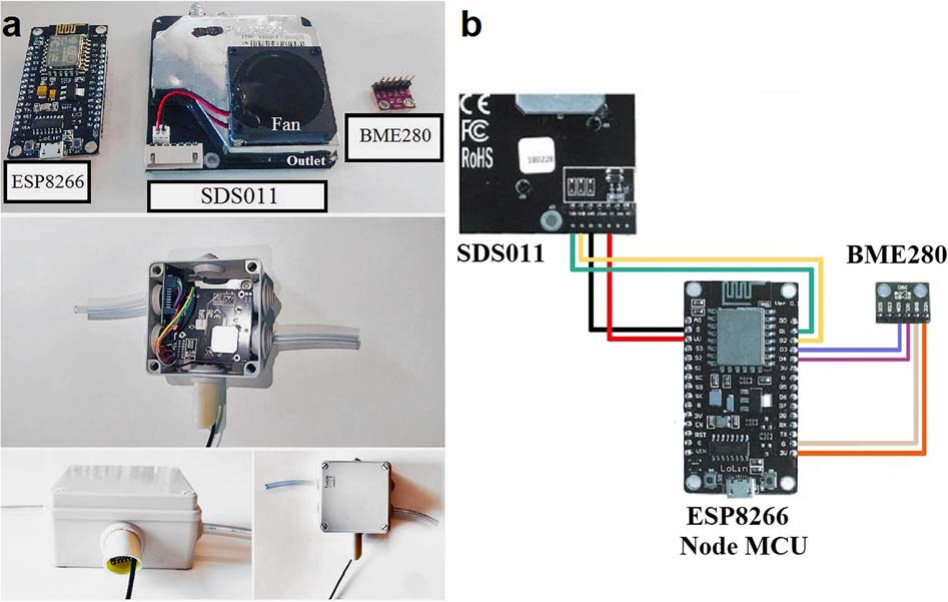
Assembly of the IoT sensor prototype. **a**) Electronic components (upper panel), enclosure and hose outlets (central panel), BME280 sensor array and power cable (bottom panel). **b**) Diagram of the connections between the individual components

The sensor prototype evaluated in this study (Fig. 1) was assembled from a low-cost PM sensor (SDS011 v1.3), an IoT Wi-Fi module (ESP8266) and an environmental sensor for air temperature and RH (BME280).

The SDS011 (Nova Fitness Ltd. Co., China) is classified as an optical sensor as it measures PM_10_ and PM_2.5_ through the principle of light scattering. A small fan produces negative pressure to create a continuous airflow from the inlet to the measuring chamber, where a laser beam radiates light to the air sample. The amount of light dispersed by the particles is detected by a photodiode, which in turn, translates the signal into electrical pulses. A microcontroller unit (MCU) analyzes these signals and calculates the PM mass concentration based on the pulse wave amplitude. According to the manufacturer, the SDS011 measures concentrations from 0.0 to 999.9 μg m^−3^ and detect particles with a minimum diameter of 0.3 μm (Nova Fitness, 2015).

The ESP8266 (Espressif Systems, China) is a low-cost Wi-Fi microchip coupled to an open hardware IoT board (Node MCU LoLin V3) and contains a USB port that also powers the module. The third component of the integrated sensor, the BME280 3.3 V (Bosch Sensortec, Germany), is a small-sized, high-resolution sensor that measures air temperature and RH.

The components were integrated using color jump wires, first connecting the ESP8266/Node MCU to the SDS011 and then to the BME280 (Fig. 1b), details of the specific connection pins are provided in the Supplementary Table 1. An IP55 junction box with lateral rubber orifices was used to house the integrated components (Fig. 1a). A Tygon® hose was inserted on the right side of the enclosure and connected to the SDS011 sensor inlet. An additional hose was inserted on the left—but not connected to any component—to allow unrestricted airflow. The USB cable and BME280 sensor were arranged inside a plastic tube at the bottom of the junction box. The Node MCU was connected to a PC and programmed using Arduino IDE software. The firmware used to configure the IoT board was downloaded from the Luftdaten project website (http://luftdaten.info).

### Field tests

During the austral winter and spring seasons of 2018, 7 units of the IoT sensor were tested in the metropolitan area of Santiago, Chile (33.4° S, 70.6° W). The typical climate of Santiago is Mediterranean, with some cold semi-arid features (Sarricolea et al. 2017). Due to its topography, Santiago experiences unfavorable conditions for the dispersion of air pollutants. The geographic and meteorological factors that affect air quality in this city have been extensively described (Rutllant and Garreaud, 1995; Schmitz, 2005; Ragsdale et al. 2013; Toro et al. 2014; Muñoz and Corral, 2017).

**Fig. 2:**
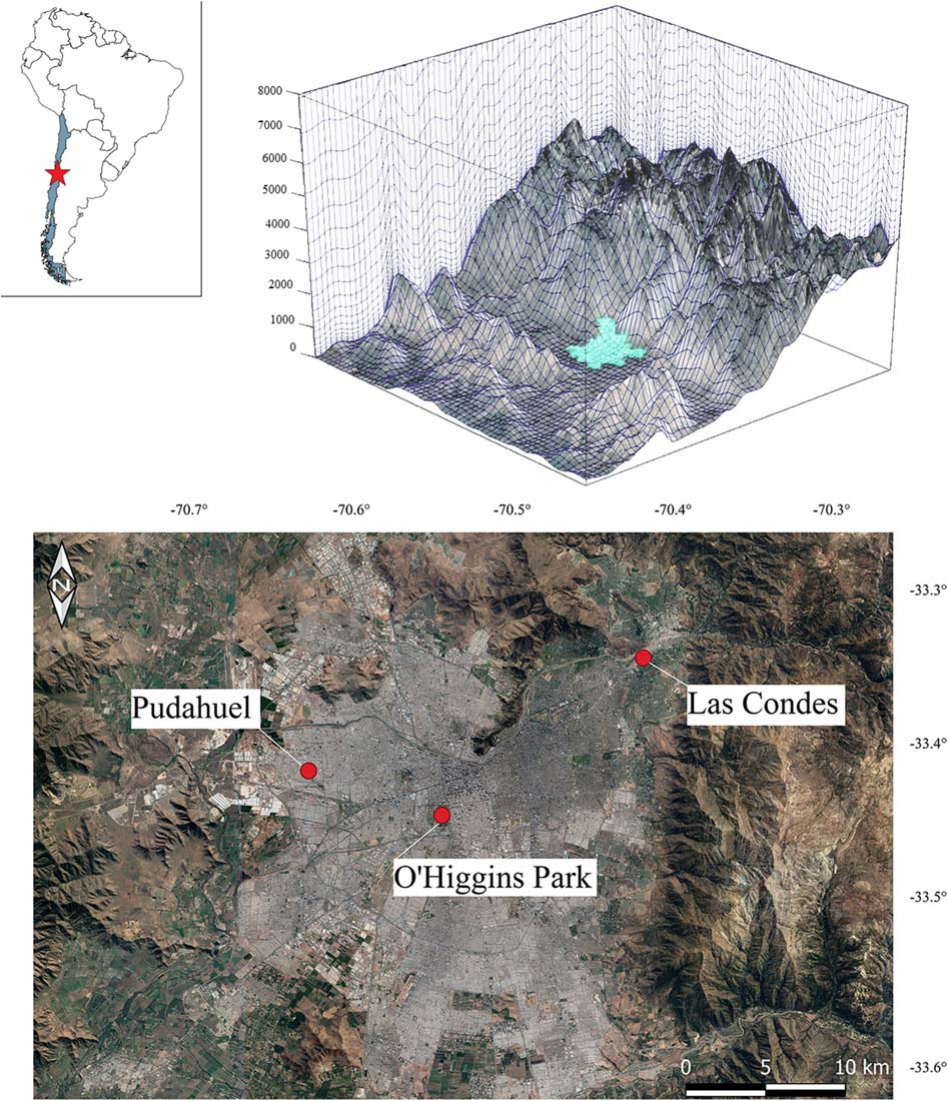
Location and physiography of Santiago, Chile (upper panel). Monitoring sites at the regulatory air quality stations

To evaluate field performance, the assembled sensors were placed at three regulatory air quality monitoring stations, Las Condes, O’Higgins Park and Pudahuel (Fig. 2), alongside U.S. Federal reference or equivalent monitors such as the BAM 1020 (Met One Inc., USA), TEOM 1400 (Thermo Scientific, USA) and filter-based sampler Partisol 2000i (Thermo Scientific, USA).

The sensors were and installed on the roof of the station (2.5 m above the ground), approximately 2 m from the monitor’s inlet, and connected to a power source using the USB cable. The Wi-Fi signal was provided by a cellphone deployed in the vicinity. The measurement time resolution was adjusted to produce one reading every 3 min. Data were automatically sent to the cloud-based storage platform belonging to the citizen science project Luftdaten (http://santiago.maps.luftdaten.info).

A week-long campaign was conducted at Las Condes station between May 24th and 31st, 2018, to evaluate reproducibility, in other words, the variation in the measurements recorded by different sensor units (*n* = 7).

Field tests were subsequently performed over longer periods of time to determine the accuracy of the sensors relative to the reference measurements. The long-term campaign was developed using a smaller number of units (*n* = 4), which were placed at Las Condes (*n* = 2) from June 1st to September 5th; O’Higgins Park (*n* = 1) from June 6th to September 7th; and Pudahuel (*n* = 1) from June 14th to September 30th.

Minor maintenance was carried out throughout the study in order to replicate normal handling of sensors by citizens as end users. Additional visits to the stations were necessary when a loss of data transmission was detected. The sensors’ databases were downloaded from the cloud-based data storage site (https://www.madavi.de/sensor/csvfiles.php) and data from the reference monitors were downloaded from the website of the public air quality monitoring network (https://sinca.mma.gob.cl).

### Statistics

A shallow scan of the database was made to discard any out-of-range values due to apparent malfunctions. The sensor readings were averaged over 1 and 24 h. The reproducibility of the 1-h averages of the 7 units arranged at Las Condes was compared through correlation analysis and normalized root-mean-squared-error (nRMSE). The nRMSE is a measure of dispersion, in which lower percentage values indicate less residual variance, and was calculated using Eq. (1), in which *y*_*i,*1_ and *y*_*i,*2_ represent the ith measurement of the pair of sensors compared and *n* is the total number of paired observations.

1 $$ nRMSE=\frac{\sqrt{\frac{1}{n}{\sum}_{i=1}^n{\left({y}_{i,1}-{y}_{i,2}\right)}^2}}{\frac{1}{2n}{\sum}_{i=1}^n\left({y}_{i,1}+{y}_{i,2}\right)} $$

The relationship between the sensor data and reference measurements during the short-term monitoring campaign at Las Condes was examined through orthogonal regression. The responses of the sensors during the long-term monitoring period were analyzed by linear regression against the 1-h and 24-h average reference concentrations reported by the BAM for PM_2.5_ and TEOM for PM_10_.

Linear regression was used to estimate the correlation between the 24-h average PM_2.5_ concentrations reported by the sensors and the filter-based samplers. Partisol 2000i, available only at Las Condes and O’Higgins Park stations, was used to collect PM_2.5_ on Teflon filters every 3 days. The filters were weighed to determine the mass concentration at the Gravimetric Laboratory of the Ministry of the Environment, Chile. The daily averages reported by the sensors were calculated from the 1-h averages. Only data from days with a completeness level of 75%, i.e., at least 18 hourly averages, were included in further analyses.

As a measure of strength of the correlations, the coefficients of determinations (*R*^2^) obtained from the linear or orthogonal regressions were calculated. The effect of air humidity on the PM sensor performance was assessed through a scatter plot colored by the RH values reported by the BME280 sensor. The average magnitude of the errors, Mean Bias Error (MBA) and Mean Absolute Error (MAE), were used to estimate the accuracy of the sensor compared to the filter-based samples. The metric expressed in μg m^−3^ was calculated as shown elsewhere (Feenstra et al., 2019).

## Results and discussion

### Inter-unit variability

**Fig. 3:**
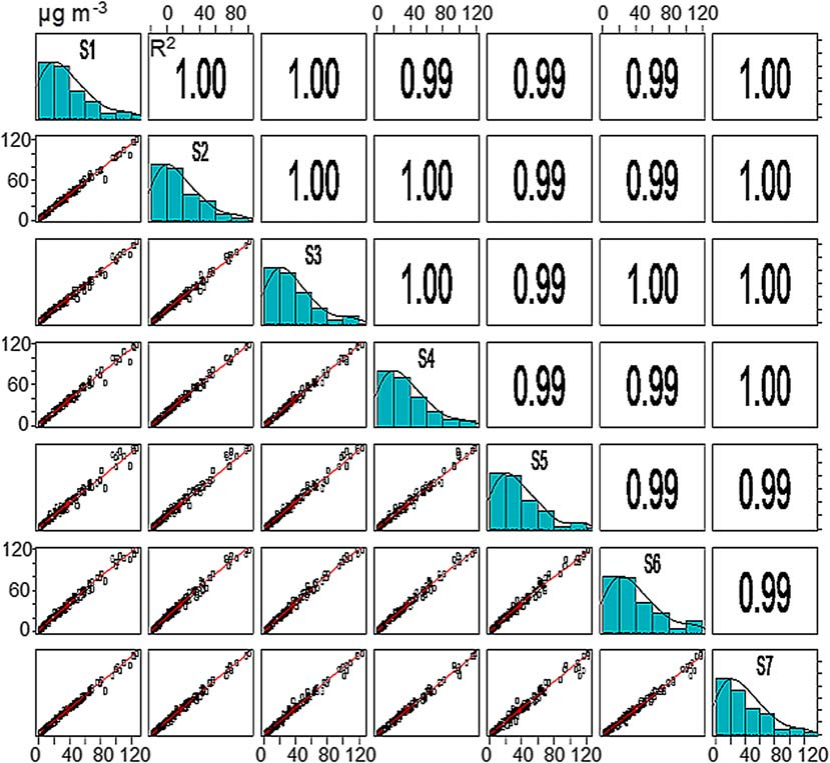
Correlation matrix for the 1-h PM_10_ average concentrations reported by different units of the SDS011 sensor (*n* = 7)

Reproducibility, or inter-unit variability, is a measure of the similarity of the data generated by different units of the same sensor model. In this study, reproducibility was calculated for the data reported by 7 units of the SDS011, the low-cost PM sensor integrated into the IoT prototype. As shown in Fig. 3, there was a high correlation between the 1-h PM_10_ average concentrations generated by all seven sensors, with *R*^2^ values ranging from 0.99 to 1. Strong linearity was also observed over the entire range of 1-h averages, with minimum and maximum average concentrations (± SD) of 1.5 (± 0.2) and 136.3 (± 15.2) μg m^−3^, respectively. The frequency distribution histogram revealed an incline in the distribution towards lower values, indicating that most of the hourly PM_10_ average concentrations recorded by the sensors were below 40 μg m^−3^.

**Fig. 4:**
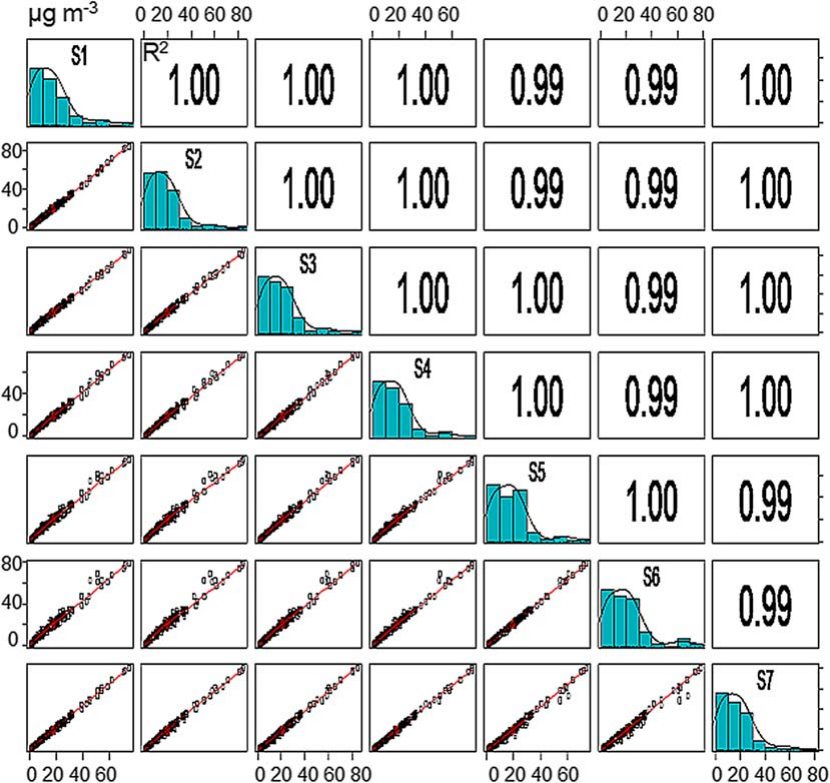
Correlation matrix for the 1-h PM_2.5_ average concentrations reported by different units of the SDS011 sensor (*n* = 7)

The correlation matrix for the 1-h average PM_2.5_ concentrations indicated high linearity and correlation between the measurements made by the 7 units (Fig. 4). Linearity was also observed over the entire concentration range, with minimum and maximum average concentrations of 0.8 (± 0.3) and 76.2 (± 6.0) μg m^−3^, respectively. Although the PM_2.5_ frequency distribution histogram was not identical to the PM_10_ histogram, some similarities were observed; for example, the higher frequency of lower concentration data.

The nRMSE was calculated to quantify the variability in the measurements between sensors. The nRMSE is expressed as a percentage; values close to zero represent lower dispersion and therefore greater reproducibility between sensors. The nRMSE ranged from 10 to 37% for PM_10_ and 9–24% for PM_2.5._ The lower nRMSE for PM_2.5_ indicates that the SDS011 unit may provide more reproducible measurements of the fine fraction than the coarse fraction.

**Fig. 5:**
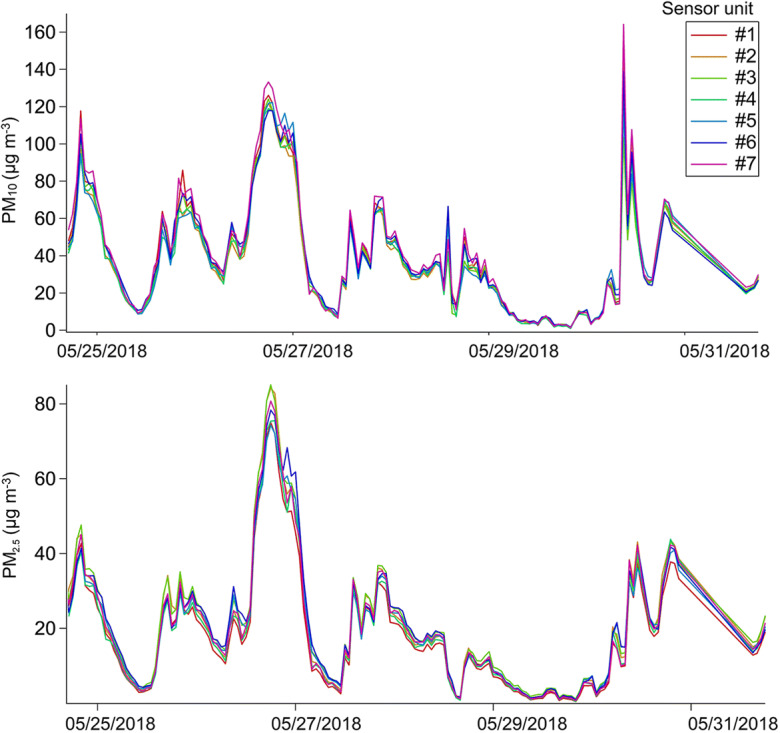
Time series of the 1-hour average concentrations reported by the different units of the SDS011 sensor deployed at Las Condes monitoring station

The time series recorded during the short-term field campaign at Las Condes monitoring station is presented in Fig. 5. The capacity of the sensors to detect the dynamics of PM_10_ and PM_2.5_ in Santiago can be inferred from the shape of the time series. For example, it was possible to observe short periods of higher PM concentrations, usually caused by the morning rush hour traffic. However, a significant increase in PM_10_ was observed towards the end of the series, together with a smaller increase in PM_2.5_ also observed. These increases may be positive artifacts due to overestimation caused by the high RH (92%) experienced during the final days of the field campaign (Sup. Fig. 1).

### Correlation between sensor and reference 1-h average concentrations

The divergence between the measurements recorded by the sensors and the reference values (BAM and TEOM) was investigated through orthogonal regression (Table 1). The correlation between the 1-h average values reported by the sensors and BAM and TEOM at Las Condes was higher for PM_2.5_ (*R*^2^ 0.67–0.72) than PM_10_ (*R*^2^ 0.40–0.47). During the measurement campaign, the 1-h PM_2.5_ average concentration reported by the sensors was approximately 5 μg m^−3^ lower than the reference 1-h average. Similarly, the sensors underestimated the reference 1-h average PM_10_ concentration by nearly 25 μg m^−3^.

**Table 1:** Correlation coefficients (*R*^2^) for the 1-h average PM_10_ and PM_2.5_ concentrations reported by the sensor replicates and the reference monitor at Las Condes station. Each concentration is the average for the one-week campaign (*n* = 150)

	PM_10_ μg m^−3^	*R*^2^	PM_2.5_ μg m^−3^	*R*^2^
Monitor				
TEOM 1400	65.1		–	
BAM 1020 (reference)	–		25.4	
Sensor				
Unit #1	40.3	0.45	18.3	0.69
Unit #2	38.6	0.41	20.7	0.67
Unit #3	38.8	0.47	21.5	0.72
Unit #4	38.0	0.44	19.1	0.68
Unit #5	39.1	0.40	20.0	0.71
Unit #6	40.5	0.45	20.9	0.71
Unit #7	42.7	0.44	20.1	0.69

The results of the correlation analysis for the long-term campaign are shown in Table 2. Linear regression analysis was performed for the 1-h averages reported by the BAM or TEOM and the sensor units arranged at the three regulatory monitoring stations. Despite power outages and loss of Wi-Fi signal affecting the data capture, acceptable levels of database completeness were achieved at each monitoring station, corresponding to 84% at Las Condes, 75% at O’Higgins Park and 87% at Pudahuel.

**Table 2:** Correlation coefficients (*R*^2^) for the 1-h average PM_10_ and PM_2.5_ concentrations reported by the sensors and the reference monitors during the long-term campaign

Station	TEOM 1400 (PM_10_)	BAM 1020 (PM_2.5_)
Las Condes		
Unit #3	0.56	0.86
Unit #4	0.53	0.84
O’Higgins Park		
Unit #2	0.24	0.51
Pudahuel		
Unit #1	0.42	0.47

As observed in the short-term campaign, a stronger correlation was observed between the sensor data and reference measurements for PM_2.5_ than for PM_10_ (Table 2). The best fit between the sensors and continuous measurements for the 1-h average PM_2.5_ concentrations was observed at Las Condes (*R*^2^ of 0.84–0.86), although the correlations between the sensor and reference PM_10_ values were considerably poorer (*R*^2^ of 0.53–0.56). The weakest correlation was observed at O’Higgins Park for the 1-h average PM_10_ concentrations (*R*^2^ 0.24).

**Fig. 6:**
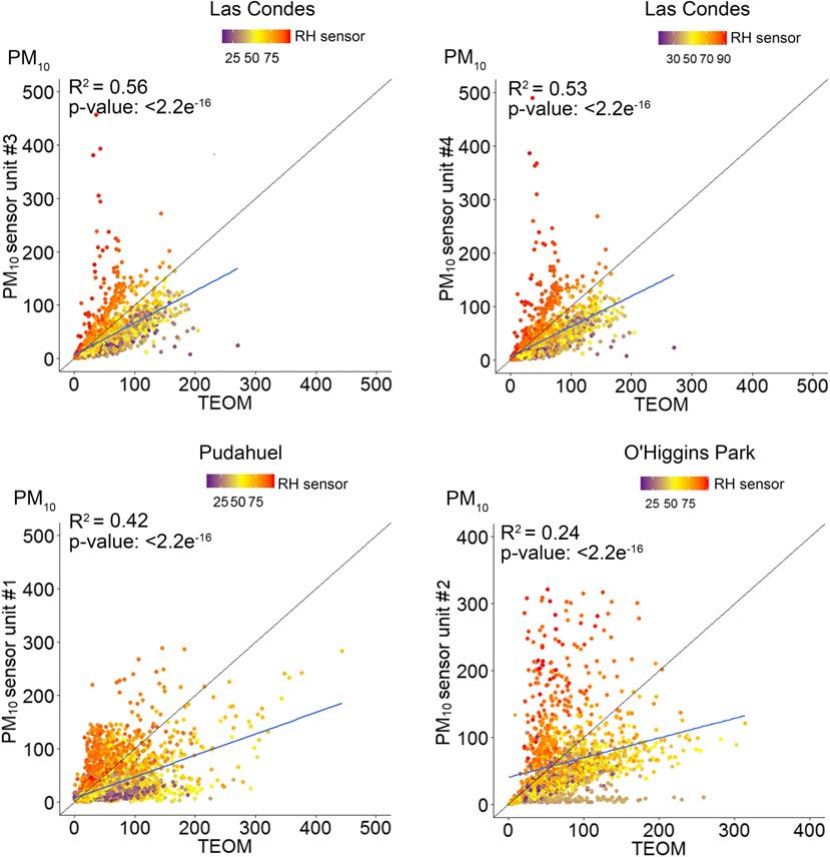
Correlation of 1-h average PM_10_ concentrations at the regulatory monitoring stations

**Fig. 7:**
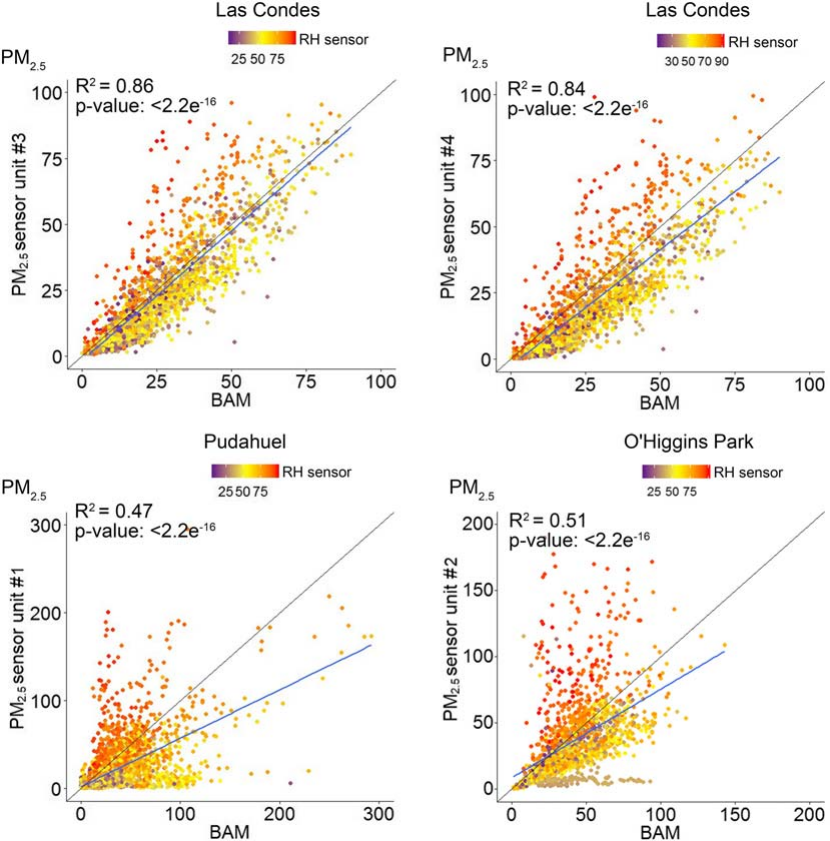
Correlation of 1-h average PM_2.5_ concentrations at the regulatory monitoring stations

A number of studies have suggested that RH is the main factor that induces overestimation by optical PM sensors, thus we investigated the accuracy of the SDS011 sensors based on 1-h average percentage RH reported by the BME280. The sensor performance suggests that was a considerable trend towards the overestimation of PM concentrations in conditions when ambient RH exceeded 75%, with a bias more intense for PM_10_ (Fig. 6) than PM_2.5_ (Fig. 7). To the greatest extent, the sensors tended to overestimate the concentrations under high humidity conditions but also tended to understate at RH below 50%.

The underestimation in Figs. 6 and 7 is represented by the values shown below the 45° diagonal line that denotes the 1:1 ratio. In all correlations, the slope of the regression was negative and under the 45° diagonal line, indicating that the sensors reported lower concentrations than the reference values most of the time. A significant underestimation reported by the sensors was observed at O’Higgins Park for PM_10_ when ambient RH was less than 40%.

### Correlation between sensor and reference 24-h average concentrations

**Fig. 8:**
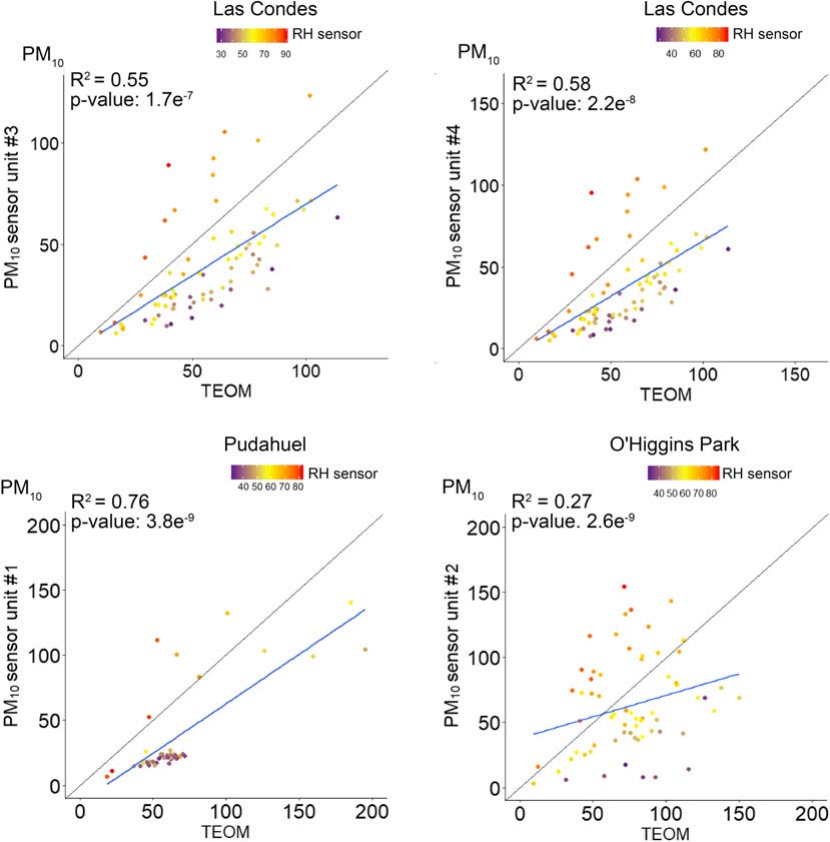
Correlation of the 24-h average PM_10_ concentrations at the regulatory monitoring stations

**Fig. 9:**
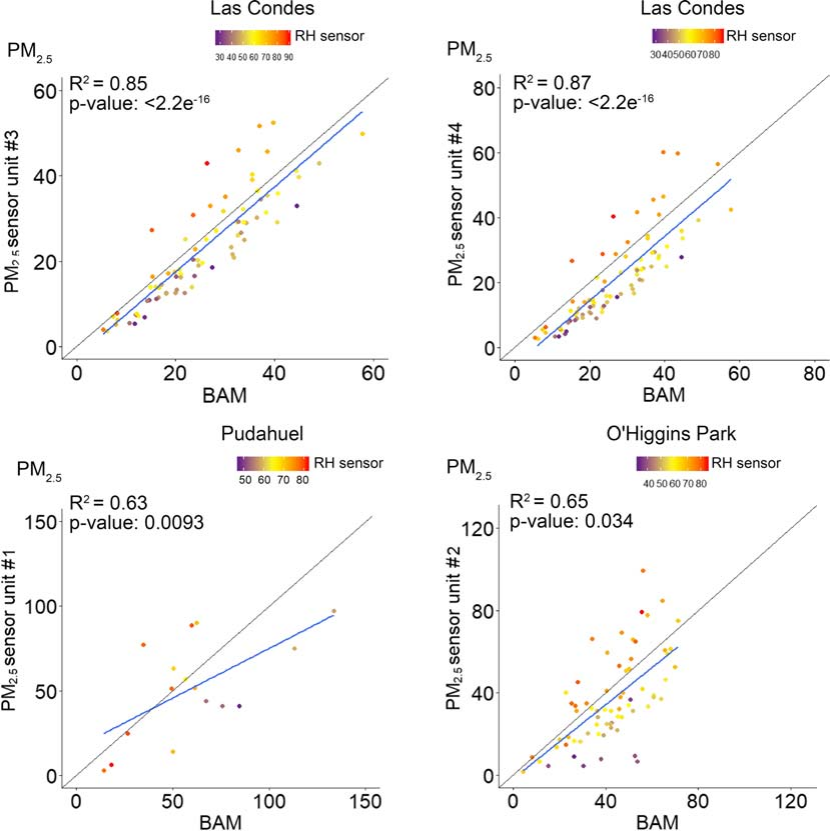
Correlation of the 24-h average PM_2.5_ concentrations at the regulatory monitoring stations

Linear correlation analysis revealed stronger correlations between the 24-h average concentrations estimated by the sensors and the reference monitors than the corresponding 1-h averages. After averaging in 24 h, the correlation for PM_10_ showed a slight increase in *R*^2^ although with significant underestimations at low RH (Fig. 8). Likewise, the 24-h correlations for PM_2.5_ averages also improved in terms of *R*^2^ and reached maximum values at Las Condes station (0.85–0.87). However, and despite the better performance, the sensor showed considerable deviations from the PM_2.5_ reference value on days with low ambient humidity (Fig. 9).

**Fig. 10:**
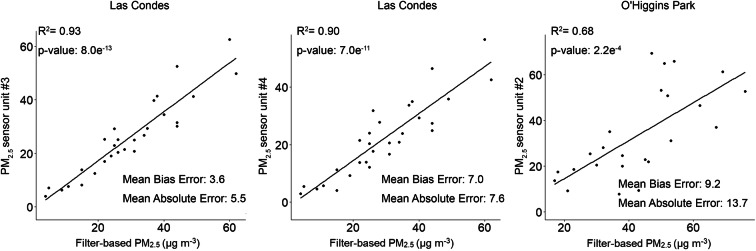
Correlation between the 24-h average PM_2.5_ concentrations reported by the sensors and the reference filter-based samplers

Figure 10 illustrates the accuracy of the 24-h measurements reported by the sensors and values generated by gravimetric analysis of the filter-based samples. The highest coefficient was observed for the units deployed at Las Condes station (Units #1 and #2), with *R*^2^ of 0.91 and 0.93. Conversely, lower correlation was observed between the reference measurements and the data captured by the sensor at O’Higgins Park (*R*^2^ 0.69). In terms of error between the reference and sensor measurements, a higher MAE was calculated for the sensor at Ohiggins Park (13.7), compared to lower MAE values determined for the sensors arranged at Las Condes (5.5 and 7.6).

Based on the comparison with the reference method of filter-based sampling, the difference in sensor performances may be explained by significant overestimations caused by the effect of ambient humidity since the environment in the O’Higgins Park experienced the highest average RH during the monitoring campaign (Sup. Fig. 2).

### Accuracy of RH sensor measurements compared to reference values

**Fig. 11:**
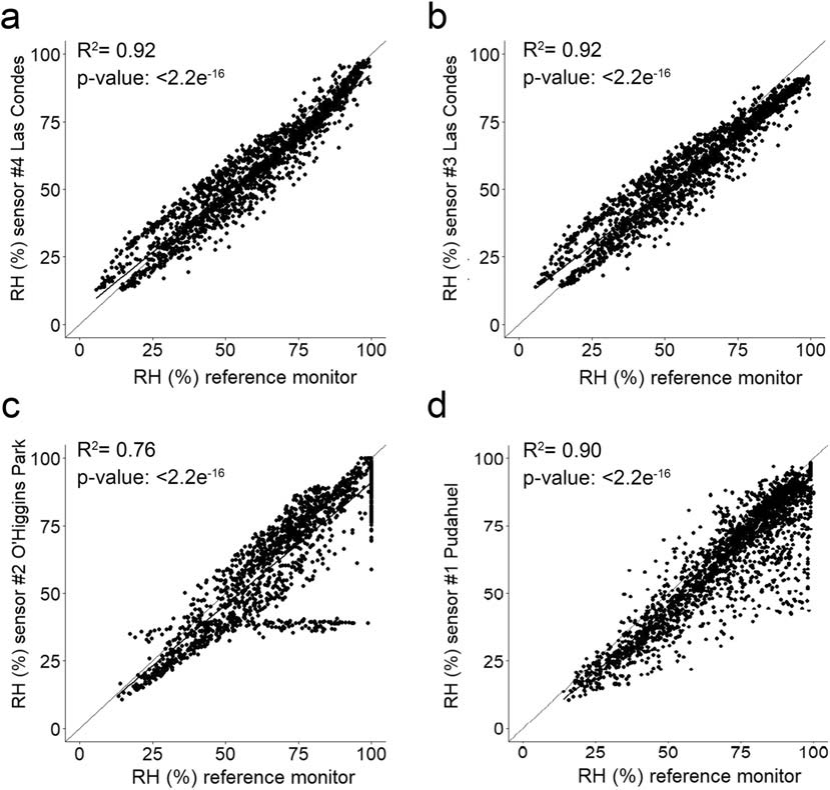
Correlation between the 24-h average RH reported by the sensors and the reference instrument

Linear regression of the 1-h RH averages was performed to compare the accuracy of the BME280 sensor against the reference instrument at the air quality stations (HMP 35A, Vaisala). As shown in Fig. 11, strong—but not fully linear—correlations were observed between the sensors and the reference measurements. The overall evaluation of the BME280 sensor indicates its performance followed the same pattern as the SDS011, that is, better correlation with reference measurements at Las Condes (*R*^2^ 0.92), and lower correlations at Pudahuel (*R*^2^ 0.90) and O’Higgins Park (*R*^2^ 0.76).

At O’Higgins Park, some stagnant measurements were recorded for 3 days (August 10–13); these measures are represented by the series that forms a horizontal line in Fig. 10c at RH 38–40%. However, no significant correlations were found in the correlations of sensor and reference monitors for PM_10_ and PM_2.5_ (Sup. Fig. 3). The latter suggests that even though the correlations shown for O’Higgins Park include the data captured during the period in which the stagnant RH measurements occurred, the size of these data points (*n* = 70) did not significantly influence the correlation obtained with the data of the whole campaign.

Also, at times when the reference instrument reported 100% RH, humidity records delivered by the sensors at O’Higgins Park and Pudahuel were observed underestimated (vertical lines of data points in Figs. 10c and d). These discrepancies suggest the RH sensors malfunctioned or external factors prevented accurate measurement of air humidity, such as obstruction caused by the enclosure or physical obstacles in the proximity of the sensors.

### Discussion of the general performance of the low-cost IoT PM sensor

The sensor prototype tested in this study estimated the 1-h PM_10_ and PM_2.5_ averages with modest (*R*^2^ ~ 0.5) and robust (*R*^2^ > 0.8) accuracy, respectively. Several field tests have shown the precision of low-cost sensors depends on the model type. Results of the 1-h correlation analysis for a variety of sensors and U.S. Federal equivalent methods are presented in Table 3. Similar to that shown in this study, better performances have been reported for the measurement of PM_2.5_ compared to PM_10_.

**Table 3:** Correlation coefficients (*R*^2^) reported in field studies for the 1-h average concentrations estimated by sensors and reference method

	Test location	PM_10_	PM_2.5_
This study	Santiago, Chile		
Nova Fitness SDS011		0.24–0.56	0.47–0.86
South Coast Air Quality Management District n.d.	Southern California, USA		
Shinyei PPD60PV		0.31–0.40	0.77–0.85
Alphasense OPC-N3		0.45–0.52	0.52–0.67
Dylos DC1700		0.15–0.18	0.58–0.68
IQAir Airvisual Pro		0.24–0.41	0.69–0.73
Crilley et al. 2018	Birmingham, UK		
Alphasense OPC-N2		0.64–0.67	0.70–0.74
Feinberg et al. 2018	Denver, Colorado, USA		
Alphasense OPC-N3		0.20–0.68	
Johnson et al. 2018	Hyderabad, India		
Shinyei PPD20V			0.81–0.86
Kelly et al. 2017	Salt Lake City, Utah, USA		
Plantower PMS1003			0.83–0.92
Badura et al. 2018	Wrocław, Poland		
Nova Fitness SDS011			0.79–0.86
Plantower PMS7003			0.83–0.89
Liu et al. 2019	Oslo, Norway		
Nova Fitness SDS011			0.55–0.71
Kuula et al. 2019	Helsinki, Finland		
Shinyei PPD60PV			0.02–0.77
Gao et al. 2015	Xi’an, China		
Shinyei PPD42NS			0.86–0.89
Feenstra et al. 2019	Riverside, California, USA		
Shinyei PM Evaluation Kit			0.73–0.75
Alphasense OPC-N2			0.38–0.67

Correlations were also better for the 24-h average concentrations than the 1-h averages, especially at Las Condes (*R*^2^ 0.84–0.86). In Santiago, Caquilpán et al. (2019) evaluated the performance of low-cost sensors models PMS3003 and PM2005 in Pudahuel and Las Condes, respectively, and reported *R*^2^ of 0.73 and 0.86 for the correlations with reference PM_2.5_ monitors. The study also indicates MAE in the range of 5–11, which decreased to 3–4 after correcting the sensor data through random forest regression. In a similar magnitude, Feenstra et al. (2019) informed MAE of 4–7 for low-cost PM_2.5_ sensors models Shinyei and Alphasense, which were tested in Riverside, California, an area with a Mediterranean climate that experienced average RH between 48 and 68%, similar to that found in Las Condes (Sup. Fig. 2). In consideration of these studies and our results, we inferred that current models of low-cost PM sensors might have a better performance for measuring PM_2.5_ in conditions of RH between 50 and 70%.

Regardless of the environment, the studies conducted to date indicate that modern low-cost sensors may be more suitable for monitoring the fine fraction than the coarse fraction. Laboratory tests have reported the SDS011 model can accurately measure particles with a d_a_ of 0.3 to 1 μm but not particles with a d_a_ greater than 5 μm (Budde et al. 2018). These findings may explain the higher reported correlations for PM_2.5_ than PM_10_ detected in this study, as well as the lowest nRMSE calculated for PM_2.5_ measurements as a metric of inter-unit variability.

Considering the data produced in parallel by different units of the IoT prototype, the inter-unit variability between the sensors is concordant and slightly better than the values reported for other optical models. In this study, the nRMSE between the SDS011 sensors ranged from 10 to 37% for PM_10_ and 9–24% for PM_2.5_. Manikonda et al. (2016) reported nRMSE for other models, such as Dylos DC1100 Pro sensor (13–46%), Sharp GP2Y1010AU0F (3–118%) and Samyoung DSM501A (22–52%). Further, Sayahi et al. (2019) assessed inter-unit variability through the nRMSE for the Plantower models PMS 1003 (63–124%) and PMS 5003 (37–57%).

Previous research has suggested that the magnitude of PM concentrations may influence reproducibility between sensors. Kuula et al. (2019) showed that inter-unit variability decreased as concentrations increased. In the same study, a magnitude of 264% of nRMSE was calculated for the Shinyei PPD60PV at low concentrations (< 4 μg m^−3^), which supports indications that performance of low-cost sensors improves as the PM concentration increases, since low concentrations are usually close to the noise level (Johnson et al. 2018; Zheng et al. 2018). Nevertheless, high concentrations could be considered potentially negative, as a higher amount of particles can cause saturation of the photodetector and lead to poor performance.

To the best of our knowledge, there no reports on the performance of the SDS011 sensor model in climates similar to Santiago, specifically, the Mediterranean type with dry summers. A few studies have assessed the performance of the SDS011 in humid subtropical climates, for example, in Thessaloniki, Greece (Genikomsakis et al. 2018) and Florence, Italy (Cavaliere et al. 2018). In both of these locations, *R*^2^ above 0.85 was observed for the correlations between the PM_2.5_ averages informed by the SDS011 and standard optical instruments. Results reported by field tests conducted in environments with higher RH due to the influence of oceanic climates, such as Wrocław, Poland, (Badura et al. 2018) and Oslo, Norway (Liu et al., 2019) suggest that SDS011 can reach modest to robust correlations in high-humidity ambient as well (*R*^2^ 0.55–0.86; Table 3).

The studies described above suggest the SDS011 is highly adaptable to different locations and climate. Differences in local ambient conditions may increase inter-sensor variability at the spatial level since areas with higher air humidity could lead to biased measurements. As an example, the better overall performance in our study was observed in sensors deployed in Las Condes, an area that presented RH between 40 and 60% and usually experiences an RH 6% lower than the one recorded at O’Higgins Park and Pudahuel (Toro et al. 2014). At sites, the sensors achieved weaker performance.

Although the intercomparison of seven SDS011 units indicated low variability between sensors, no corrections of raw data were made, which can be considered a limitation of this study. However, in addition to the low inter-sensor variability, the greater correlation found in the comparisons with the reference filter-based sampling indicates that the evaluated sensors can accurately capture the 24-h average of PM_2.5_ concentration with reduced spatial variation at the city scale.

Other limitations of the current version of the sensor must be recognized, including the difficulty of detecting black carbon due to the low light scattering ability of these particles. Also, the lack of accuracy in measurement of PM_10_ concentrations and, to a lesser extent, PM_2.5_, may reflect the limitations of this sensor technology, such as the absence of the drying system present in reference monitors (Budde et al. 2018). Furthermore, our evaluation revealed discrepancies between the BME280 sensor and the reference hygrometer, suggesting that the design of the sensor enclosure may impose additional limitations.

## Conclusions

An IoT prototype for air quality monitoring was assembled by integrating a low-cost PM sensor (SDS011), a temperature and RH sensor (BME280) and an IoT module (ESP8266). The IoT component conferred the ability to transmit data in real time to the cloud-based storage platform. During the winter and spring of 2018, the IoT prototype was evaluated at three regulatory monitoring stations in Santiago, Chile. The field tests revealed low inter-unit variability and good linearity with reference data, though the sensors had a limited capacity to estimate the correct concentrations of airborne particles. The sensor performed reasonably in terms of 24-h average PM_2.5_ concentrations; however, considerable bias was observed in the 1-h average measurements, including overestimation when RH exceeded 75% and underestimation when RH was under 50%. Overall, the performance of the sensor was adequate for PM_2.5_, but not for PM_10_. The BME280 sensor data exhibited a good fit and precision with the reference RH measurements. However, some underestimation of high humidity was detected at two monitoring stations, indicating possible interference with the enclosure. Future research on this prototype is required to address and reduce biases using calibration methods that incorporate the RH variable. Overall, we conclude that the SDS011 sensor is suitable for citizen science projects, and with some refinements, could eventually be suitable for expansion of the current air quality monitoring network in Santiago.

## Electronic supplementary material

ESM 1 (PDF 164 kb)
