# Supplementary material for: Field performance of a low-cost sensor in the monitoring of particulate matter in Santiago, Chile
Source: Environ Monit Assess. 2020 Feb 10;192(3):171. doi: 10.1007/s10661-020-8118-4 (PMC7010625; doi:10.1007/s10661-020-8118-4)
Supplement: Supplementary file 1 — (PDF 164 kb) [file 10661_2020_8118_MOESM1_ESM.pdf]

Supplementary Material

Field performance of a low-cost IoT sensor in the monitoring of particulate matter in Santiago, Chile

Matías Tagle[1], Francisca Rojas[1], Felipe Reyes[1], Yeanice Vásquez[1], Fredrik Hallgren[2], Jenni Linden[2], Dimitar Kolev[3], Ågot Watne[4], Pedro Oyola[1*]

Supplementary Table 1. Connection pins between the different components of the IoT sensor.

Wire color	SDS011	ESP8266/ Node MCU	BME280	Function
Green	TXD	D1		Connects SDS011 to ESP8266/Node MCU
Yellow	RXD	D2		Connects ESP8266/Node MCU to SDS011
Black	GND	G		Connects to ground
Red	5 v	VU		Energizes with 5 volts
Orange		3 V	VCC	Energizes with 3 volts
White		G	GND	Connects to ground
Purple		D4	SCL	Sends data by I2C protocol
Blue		D3	SDA	Sends data by I2C protocol

* Corresponding author. Tel: (562) 2247 9650

E-*mail address*: poyola@cmmolina.cl

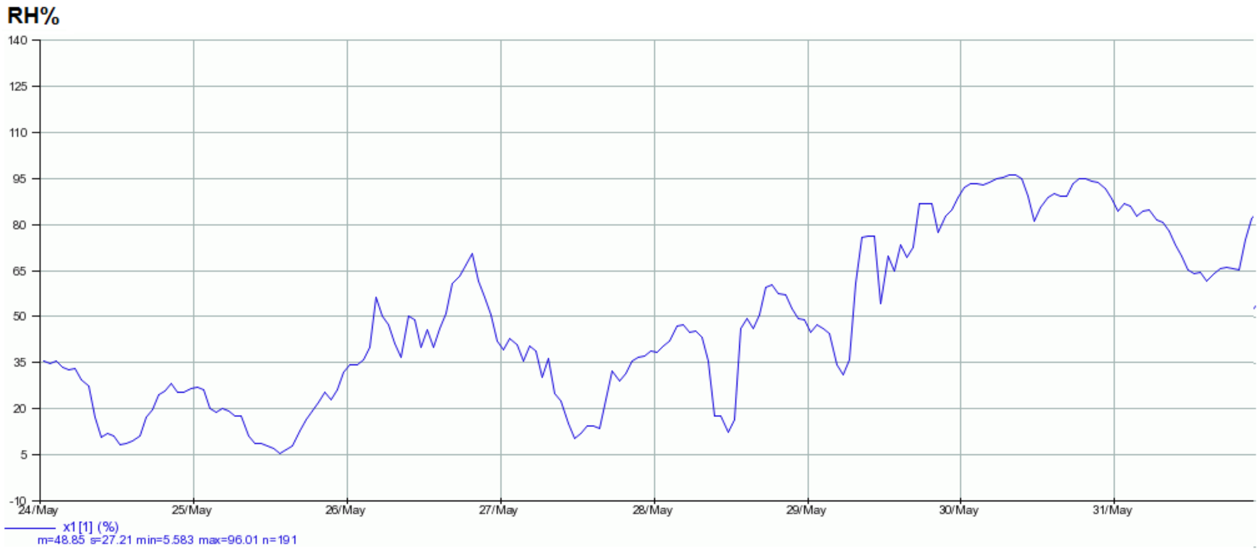

Supplementary Figure 1. Relative humidity at the Las Condes regulatory station during the short-term monitoring campaign.

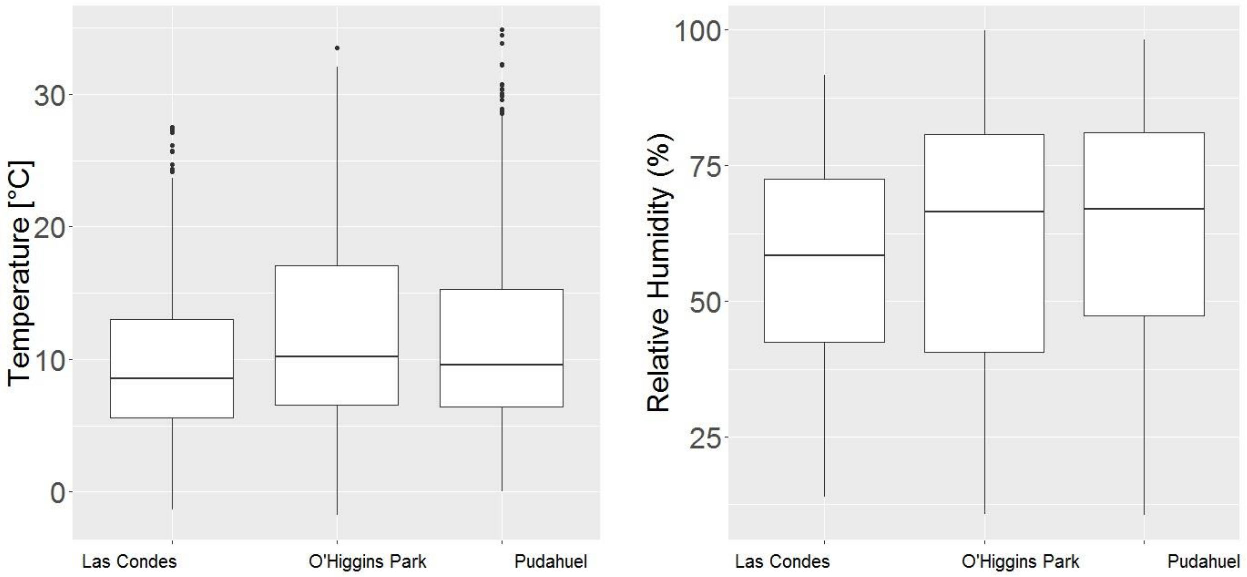

Supplementary Figure 2. Temperature and Relative Humidity at the three regulatory monitoring stations during the long-term monitoring campaign.

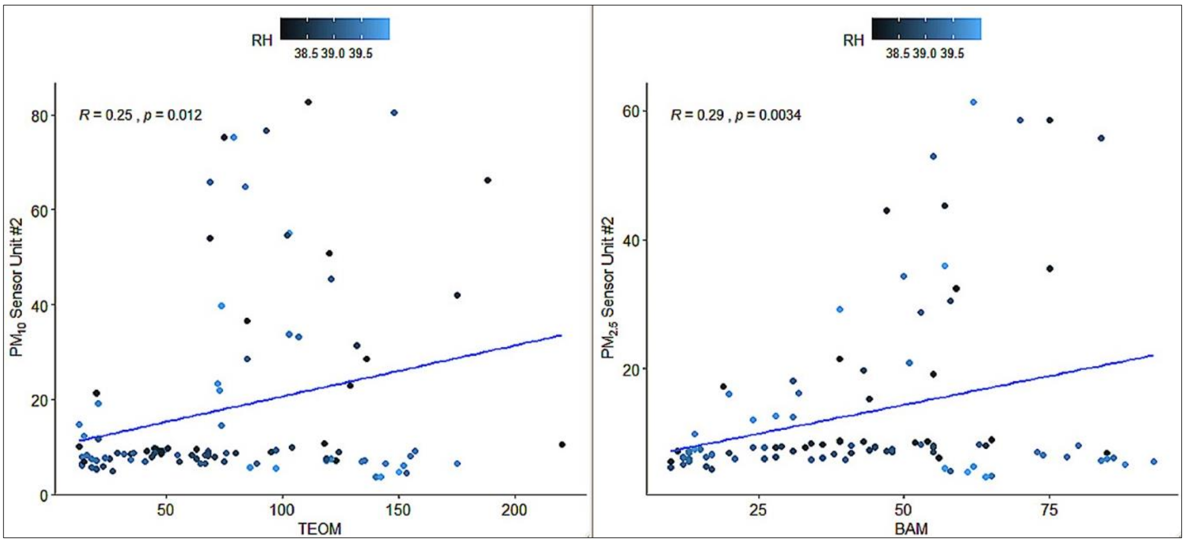

Supplementary Figure 3. Correlation of the 1-h averages reported by the SDS011 sensor and the reference monitor (TEOM and BAM) for RH range 38-40% informed by the BME280 sensor at O'Higgins Park.
